# Metabolome-wide Mendelian randomization reveals causal effects of betaine and N-acetylornithine on impairment of renal function

**DOI:** 10.3389/fnut.2024.1371995

**Published:** 2024-04-24

**Authors:** Yuqing Liu, Lilu Ling, Yue Shen, Xiao Bi

**Affiliations:** ^1^Department of Nephrology, Tongji Hospital, School of Medicine, Tongji University, Shanghai, China; ^2^Division of Nephrology, Shanghai Ninth People’s Hospital, School of Medicine, Shanghai Jiaotong University, Shanghai, China

**Keywords:** renal function, genetically determined metabolites, betaine, N-acetylornithine, Mendelian randomization

## Abstract

**Background:**

Chronic kidney disease (CKD) is a common public health problem, which is characterized as impairment of renal function. The associations between blood metabolites and renal function remained unclear. This study aimed to assess the causal effect of various circulation metabolites on renal function based on metabolomics.

**Methods:**

We performed a two-sample Mendelian randomization (MR) analysis to estimate the causality of genetically determined metabolites on renal function. A genome-wide association study (GWAS) of 486 metabolites was used as the exposure, while summary-level data for creatinine-based estimated glomerular filtration rate (eGFR) or CKD occurrence were set the outcomes. Inverse variance weighted (IVW) was used for primary causality analysis and other methods including weight median, MR-egger, and MR-PRESSO were applied as complementary analysis. Cochran Q test, MR-Egger intercept test, MR-PRESSO global test and leave-one-out analysis were used for sensitivity analysis. For the identified metabolites, reverse MR analysis, linkage disequilibrium score (LDSC) regression and multivariable MR (MVMR) analysis were performed for further evaluation. The causality of the identified metabolites on renal function was further validated using GWAS data for cystatin-C-based eGFR. All statistical analyses were performed in R software.

**Results:**

In this MR analysis, a total of 44 suggestive associations corresponding to 34 known metabolites were observed. After complementary analysis and sensitivity analysis, robust causative associations between two metabolites (betaine and N-acetylornithine) and renal function were identified. Reverse MR analysis showed no causal effects of renal function on betaine and N-acetylornithine. MVMR analysis revealed that genetically predicted betaine and N-acetylornithine could directly influence independently of each other. The causal effects of betaine and N-acetylornithine were also found on cystatin-C-based eGFR.

**Conclusion:**

Our study provided evidence to support the causal effects of betaine and N-acetylornithine on renal function. These findings required further investigations to conduct mechanism exploration and drug target selection of these identified metabolites.

## Introduction

Chronic kidney disease (CKD) is a common public health problem which affects up to 10% of the adults population worldwide (1). It is often characterized by decreased glomerular filtration rate (GFR) which can be estimated from the serum creatinine or cystatin-C level. Various factors play a role in renal function, including cytokines, metabolites, vascular regulation and renal innervation (2). Although many underlying causes such as hypertension, diabetes and drugs with renal toxicity are known, CKD etiology still remains at least partially unclear, which deserves further investigations.

So far, metabolomics is used widely to detect small molecules in serum, urine or other biosamples, which provides a novel insight for the mechanistic understanding of diseases (3). Metabolites, produced by upstream genes and proteins, may act as important nutrients or harmful products for human health. Previous studies indicated some gut microbiota derived metabolites such as arginine (4), butenoylcarnitine (5) and tryptophan metabolism (6) may participate in the development of chronic kidney disease, underlying the associations between blood metabolites and renal function. Identification of blood metabolites beneficial to renal function may provide novel insight into renal nutrition treatment.

Mendelian randomization (MR) analysis is a novel method using genetic variants to estimate causal effects of exposure on clinical outcomes. It is less likely to be affected by confounding factors and reverse causation, therefore overcoming the limitations of conventional observational studies (7). Recently, genome-wide association studies (GWAS) were extended to metabolic phenotypes which determined genetic variants for metabolite traits including non-targeted metabolomics (8). This allows researchers to analyze causal interaction of blood metabolites on diseases.

In this study, we performed metabolome-wide MR analysis based on GWAS datasets of 486 metabolites and meta-analysis of renal function from CKD Genetic Consortium and UK Biobank. We aimed to assess causal effects of metabolites on renal function and to identify potential metabolites that could increase or decrease the risk of renal function impairment, which may become nutritional treatment targets of CKD.

## Materials and methods

### Study design

**Figure 1:**
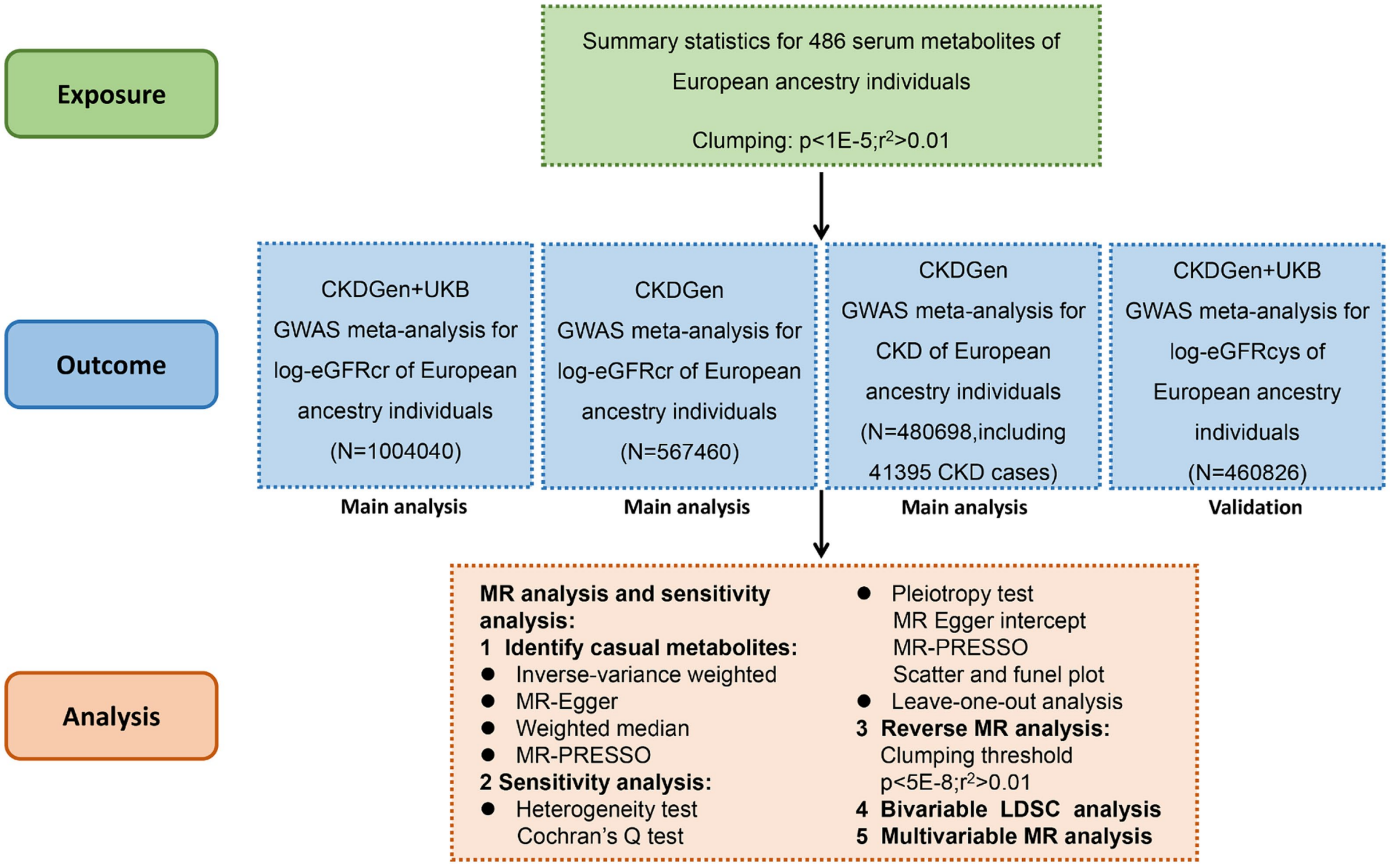
Flowchart of the study design.

We systematically assessed the causal association between human blood metabolites and renal function using two-sample MR analysis. The instrumental variables (IVs) should comply with 3 fundamental assumptions (9):(1) the genetic instruments are supposed to have direct associations with exposure; (2) the genetic instruments are supposed to be unrelated with the outcome (i.e., eGFR or CKD in this study) or its confounding factors; (3) the genetic instruments does not affect the outcome except potentially via the exposures of interest. The flowchart of this study was demonstrated in Figure 1.

### GWAS data for human blood metabolites as exposures

The summary data for blood metabolites were obtained from the metabolomics GWAS server.^1^ In brief, it was a GWAS meta-analysis consisting of two European cohorts (TwinsUK and KORA cohorts) with 7,824 European adult individuals (10). 486 metabolites were identified and approximately 2.1 million SNP were included in this dataset. Among the 486 metabolites, 309 were known metabolites which could be classified into eight biochemical classes (peptides, nucleotides, amino acids, energy, cofactors and vitamins, lipids, carbohydrates, and xenobiotics). Another 177 were unknown metabolites with undetermined chemical identity. More detailed information could be obtained from the previous study. In this study, we adopted the genetic associations and pathway information of 309 known metabolites for MR analysis so as to identify the metabolites potentially related with renal function impairment.

### Selection of instrumental variables

Single nucleotide polymorphisms (SNPs) selected as IVs should satisfy the three fundamental assumptions. For each metabolite, SNPs were extracted at a relaxed association thresholds at *p* < 1 × 10^−5^. Linkage-disequilibrium threshold was set r^2^ < 0.01 within a window of 500 kb in the European 1,000 Genomes Project Phase 3 reference panel. Then we calculated F statistic of each SNP and excluded SNPs with F statistic below 10 to avoid weak instruments bias (11). Palindromic SNPs or SNPs with missing values were removed in the process of harmonizing (12). We also checked IVs for metabolites at the Phenoscanner V2 website^2^ to exclude SNPs associated with renal function or CKD trait (*p* < 1 × 10^−5^).

### GWAS data for renal function outcome

The CKDGen consortium provides a publicly available database for GWAS meta-analysis of renal function.^3^ eGFR values are commonly used for the evaluation of renal function. We used three eGFR outcome datasets as follows for main analysis: (1) the meta-analysis of the CKDGen and UKB data from 2021 using creatinine-based log-eGFR values; (2) the phase 4 CKDGen study from 2019 using creatinine-based log-eGFR values; (3) CKDGen study from 2019 including CKD cases and controls, in which CKD was define as creatinine-based eGFR<60 mL/min/1.73m^2^. The population of the three datasets were European ancestry. To enhance the reliability of the results, we also adopted an additional dataset from CKDGen and UKB from 2021 using cystatin-C-based log-eGFR values. The population of this dataset was also European ancestry. This may help to make our conclusions more convincible. More detailed information could be found in the previous studies (13, 14).

## Statistical analysis

### Mendelian randomization analysis

The causal interactions between blood metabolites and renal function were preliminarily assessed by standard inverse variance weighted (IVW) method. If *p* value of IVW was less than 0.05 in either of the datasets for main analysis, the metabolite was selected as candidate metabolite. Then MR-PRESSO was applied to detect outliers of the instrumental variables. After remove of the outliers (or keeping all SNPs as instrumental variables if no outliers were found), we estimated the causal effect of candidate metabolites on renal function using four methods including IVW, MR-Egger, Weighted Median and MR-PRESSO test. Several sensitivity analyses were performed for validation of validity of IVs. The Cochran’s Q test and associated *p*-values were calculated to examine the presence of heterogeneity and pleiotropy. If the null hypothesis was rejected, random-effect IVW was performed instead of fixed-effect IVW. MR-Egger intercept, MR-PRESSO global test and the funnel plot was applied to estimated horizontal pleiotropy. For all the sensitivity analysis, a two-tailed *p* < 0.05 was considered statistically significant. Leave-one-out analysis was performed to test if the causal associations were dramatically affected by individual SNPs. To exclude the possibly bi-directional associations between metabolites and renal function, we also performed reverse MR analysis regarding the three renal function datasets and identified metabolites, respectively.

### Genetic correlation analysis

To estimate the overall genetic correlation between blood metabolites and renal function, we conducted a bivariable linkage disequilibrium score (LDSC) regression for identified metabolites in the MR analysis (15).

### Multivariable MR analysis

To further confirm the direct effects of each identified metabolite on renal function (independent of each other), multivariable MR analysis (MVMR) was applied. MVMR could correct for interactions of genetic variant between exposures by incorporating multiple exposures that may interact with each other (16). MVMR was performed using IVW, MR-Lasso Feature and MR-PRESSO. The IVW method of multivariate MR is to regress all exposed SNPs with the outcome, weighting for the inverse variance of the outcome. MR-Lasso could exclude collinearity of IVs. MR-PRESSO could remove outliers to correct for the pleiotropy of IVs.

### Analysis software

The analyses were carried out using R (version 4.3.0). The R packages included TwoSampleMR, MRPRESSO, ggplot2, pheatmap, ldscr and forest plot.

## Results

### Causal effects of metabolites on renal function from three European ancestry datasets

**Figure 2:**
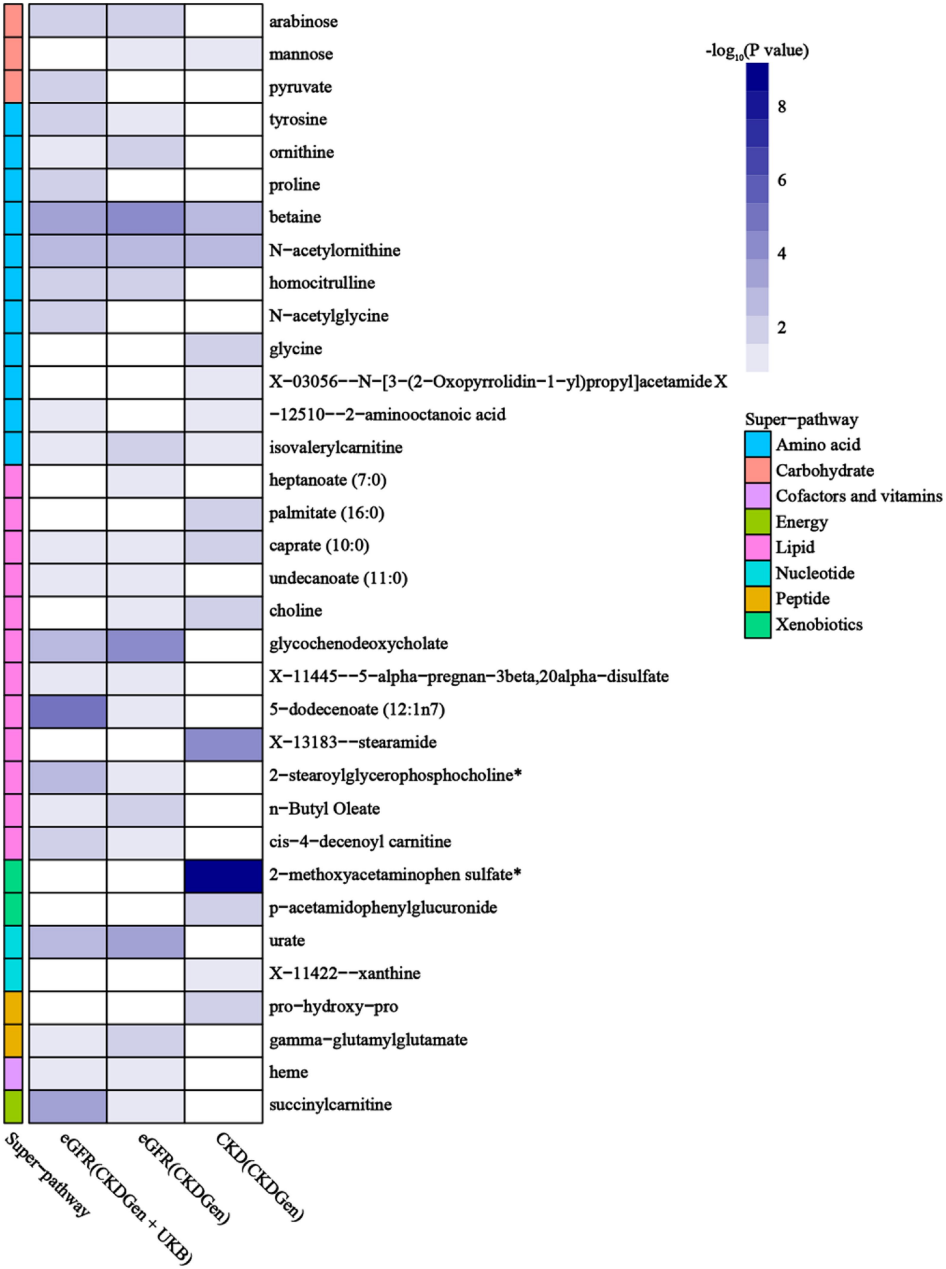
Mendelian randomization associations of known metabolites with various renal function phenotype (derived from the fixed-effect or random-effect IVW analysis according to the Cochran’s Q test). IVW, inverse-variance weighted.

**Figure 3:**
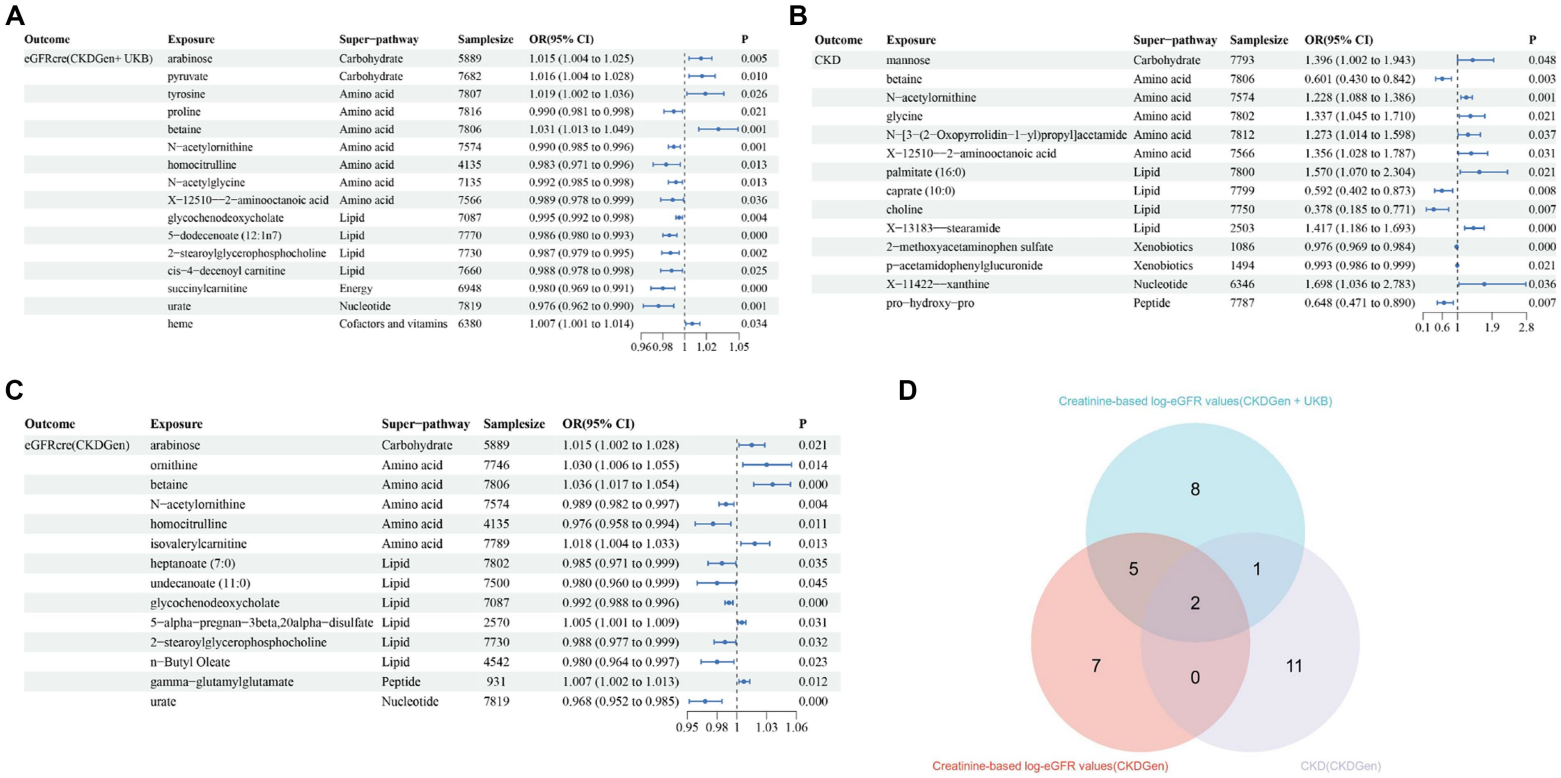
The causal estimates of the candidate metabolites on various renal function phenotype using IVW method. **(A)** Candidate metabolites with creatinine-based eGFR from CKDGen and UKB; **(B)** candidate metabolites with creatinine-based eGFR from CKDGen; **(C)** candidate metabolites with CKD from CKDGen; **(D)**Venn diagram showing the two metabolites (betaine and N-acetylornithine) were significantly correlated with renal function phenotype from all three datasets.

Using selected IVs, we estimated causal effects of 309 metabolites with known structures and functions on renal function. After remove of outliers, we identified a total of 44 suggestive associations (*p* < 0.05, corresponding to 34 known metabolites) as shown in Figure 2 and Supplementary Table S1. Details of IVW, sample size and *p* value were demonstrated in Figures 3A–C. Using Venn diagram (shown in Figure 3D) we found that two metabolites showed significant causal interactions with renal function phenotype from all three datasets. They were betaine and N-acetylornithine OR (95% CI) = 1.031 (1.013–1.049), *p* = 0.00051 for betaine and OR (95% CI) = 0.990 (0.985–0.996), *p* = 0.0012 for N-acetylornithine in analysis with Creatinine-based log-eGFR values provided by CKDGen and UKB; OR (95% CI) = 1.036 (1.017–1.054), *p* = 0.00010 for betaine and OR (95% CI) = 0.989 (0.982–0.997), *p* = 0.0041 for N-acetylornithine in analysis with Creatinine-based log-eGFR values provided by CKDGen; OR (95% CI) = 0.601 (0.430–0.842), *p* = 0.0031 for betaine and OR (95% CI) = 1.228(1.088–1.386), *p* = 0.00091 for N-acetylornithine in analysis with CKD outcome provided by CKDGen. Details of instrumental variables were shown in Supplementary Table S2.

### Sensitivity analysis

Sensitivity analysis was performed to assess the robustness of the causal effects. The results of sensitivity analysis for betaine and N-acetylornithine were shown in Table 1. For the IVW method, *p* value of Cochran’s Q statistic for betaine was 0.0013 in analysis with creatinine-based log-eGFR values provided by CKDGen and UKB and 0.015 in analysis with creatinine-based log-eGFR values provided by CKDGen and random-effect IVW was performed instead of fixed-effect IVW. Other *p* values of Cochran’s Q statistic did not show statistically significant. We screened out horizontal pleiotropy in all associations by MR-Egger’s intercept term and MR-PRESSO’s global test.

**Table 1:** Sensitivity analysis of the identified metabolites on renal function phenotype.

Outcome	Metabolite	SNP number	Method	*p* value	OR (95% CI)	
Creatinine-based log-eGFR values (CKDGen + UKB)	Betaine	18	IVW(random)	0.00051	1.031 (1.013–1.049)	P_Q_IVW_ = 0.0013
		18	WM1	0.0013	1.031 (1.012–1.051)	
		18	WM2	0.0061	1.036 (1.013–1.059)	
		18	MR Egger	0.20	1.026 (0.988–1.067)	P_intercept_Egger_ = 0.79
		18	MR Presso	0.0029	1.031 (1.013–1.049)	P_global test_ = 0.065
Creatinine-based log-eGFR values (CKDGen + UKB)	N-acetylornithine	20	IVW(fixed)	0.0012	0.990 (0.985–0.996)	P_Q_IVW_ = 0.58
		20	WM1	0.073	0.992 (0.984–1.001)	
		20	WM2	0.29	0.991 (0.976–1.007)	
		20	MR Egger	0.56	0.995 (0.980–1.011)	P_intercept_Egger_ = 0.53
		20	MR Presso	0.0031	0.990 (0.985–0.996)	P_global test_ = 0.57
Creatinine-based log-eGFR values (CKDGen)	Betaine	16	IVW(fixed)	0.00010	1.036 (1.017–1.054)	P_Q_IVW_ = 0.10
		16	WM1	0.0069	1.033 (1.009–1.057)	
		16	WM2	0.082	1.031 (0.998–1.070)	
		16	MR Egger	0.23	1.027 (0.986–1.070)	P_intercept_Egger_ = 0.66
		16	MR Presso	0.0015	1.036 (1.017–1.054)	P_global test_ = 0.15
Creatinine-based log-eGFR values (CKDGen)	N-acetylornithine	21	IVW(fixed)	0.0041	0.989 (0.982–0.997)	P_Q_IVW_ = 0.65
		21	WM1	0.046	0.989 (0.979–0.999)	
		21	WM2	0.30	0.991 (0.973–1.008)	
		21	MR Egger	0.82	0.998 (0.978–1.018)	P_intercept_Egger_ = 0.39
		21	MR Presso	0.0055	0.989 (0.983–0.996)	P_global test_ = 0.63
CKD (CKDGen)	Betaine	22	IVW(fixed)	0.0031	0.601 (0.430–0.842)	P_Q_IVW_ = 0.46
		22	WM1	0.15	0.697 (0.426–1.140)	
		22	WM2	0.40	0.768 (0.424–1.393)	
		22	MR Egger	0.56	0.785 (0.353–1.745)	P_intercept_Egger_ = 0.48
		22	MR Presso	0.0073	0.601 (0.430–0.841)	P_global test_ = 0.52
CKD (CKDGen)	N-acetylornithine	25	IVW(fixed)	0.00091	1.228 (1.088–1.386)	P_Q_IVW_ = 0.17
		25	WM1	0.0021	1.250 (1.084–1.441)	
		25	WM2	0.0040	1.236 (1.085–1.408)	
		25	MR Egger	0.033	1.247 (1.030–1.510)	P_intercept_Egger_ = 0.83
		25	MR Presso	0.0029	1.228 (1.088–1.386)	P_global test_ = 0.26

WM1, Weighted Median; WM2, Weighted Mode.

**Figure 4:**
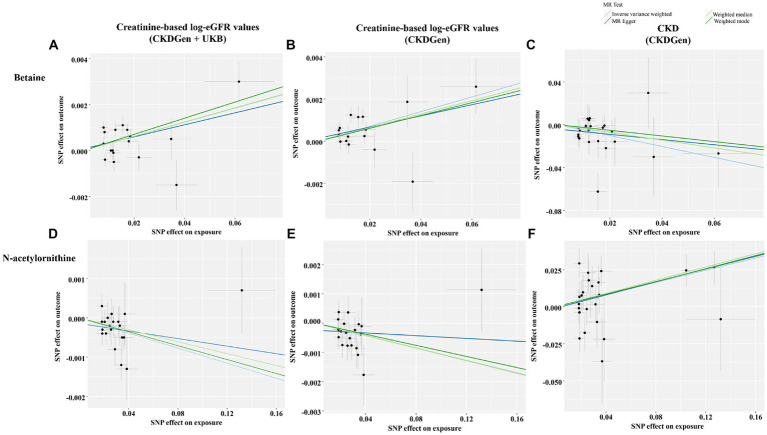
Scatter plots showing the genetic associations of the identified metabolites with various renal function phenotype. **(A)** Betaine on creatine-based eGFR from CKDGen and UKB; **(B)** betaine on creatine-based eGFR from CKDGen; **(C)** betaine on CKD phenotype; **(D)** N-acetylornithine on creatine-based eGFR from CKDGen and UKB; **(E)** N-acetylornithine on creatine-based eGFR from CKDGen; **(F)** N-acetylornithine on CKD phenotype.

**Figure 5:**
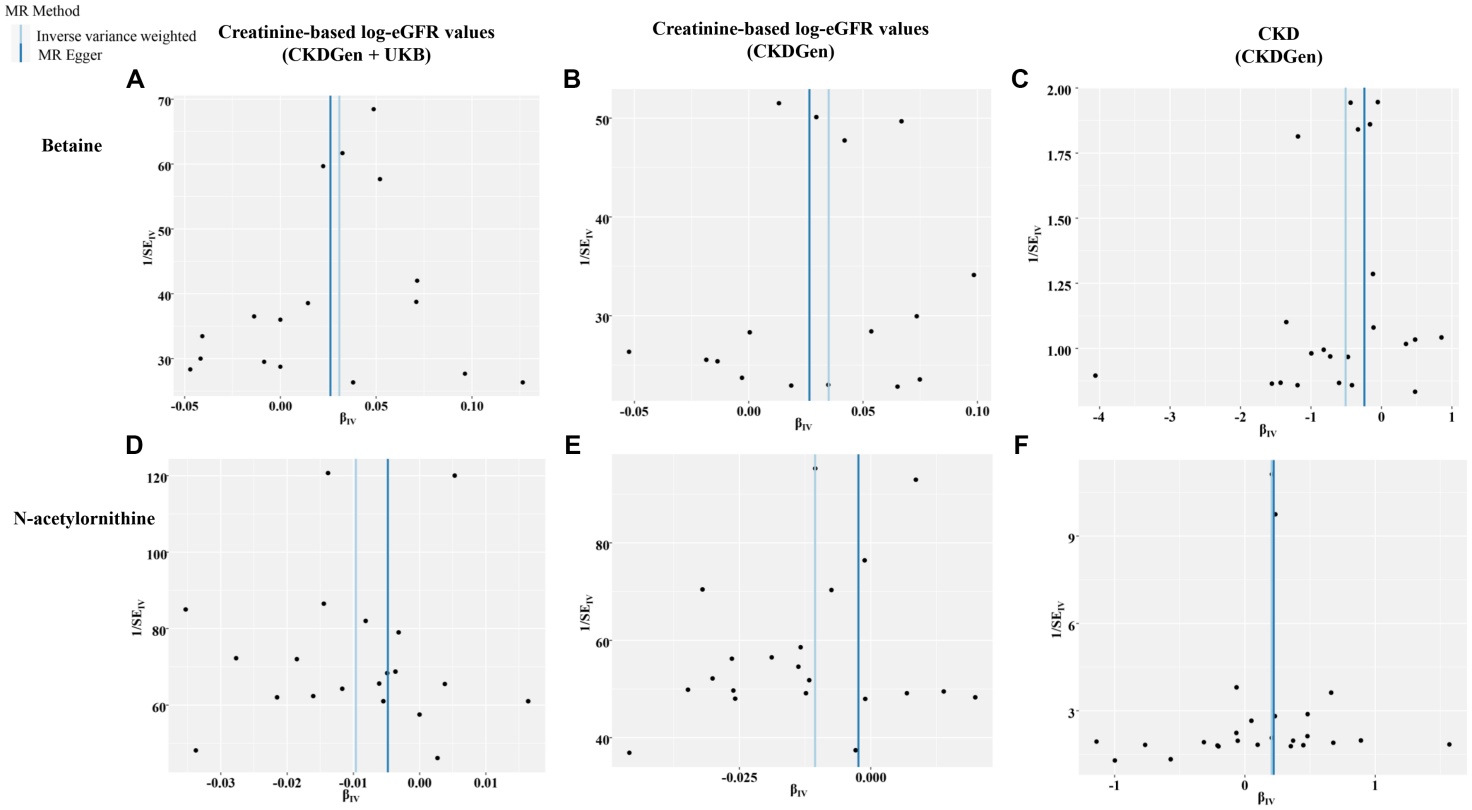
Funnel plots representing IVs for each significant causal association between the identified metabolites and renal function phenotype. **(A)** Betaine on creatine-based eGFR from CKDGen and UKB; **(B)** betaine on creatine-based eGFR from CKDGen; **(C)** betaine on CKD phenotype; **(D)** N-acetylornithine on creatine-based eGFR from CKDGen and UKB; **(E)** N-acetylornithine on creatine-based eGFR from CKDGen; **(F)** N-acetylornithine on CKD phenotype.

**Figure 6:**
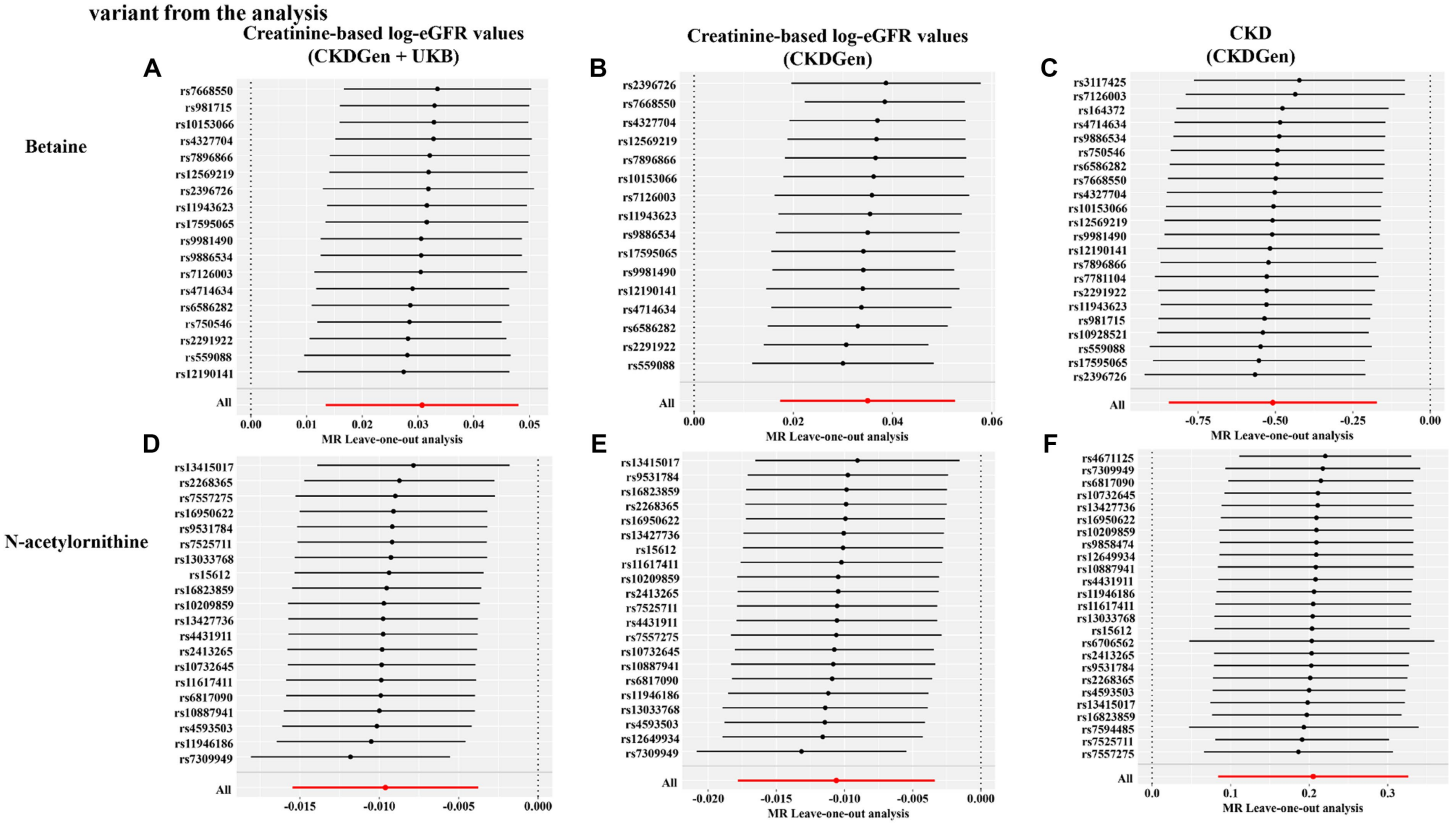
Leave-one-out sensitivity analysis showing the causal effect of the identified metabolites on renal function phenotype excluding that certain variant from the analysis. **(A)** Betaine on creatine-based eGFR from CKDGen and UKB; **(B)** betaine on creatine-based eGFR from CKDGen; **(C)** betaine on CKD phenotype; **(D)** N-acetylornithine on creatine-based eGFR from CKDGen and UKB; **(E)** N-acetylornithine on creatine-based eGFR from CKDGen; **(F)** N-acetylornithine on CKD phenotype.

In addition, scatter plots (Figure 4) and funnel plots (Figure 5) were also applied to rule out the possibility of potential outliers and horizontal pleiotropy for betaine and N-acetylornithine. Leave-one-out analysis was used to avoid the heavy influence of a single SNP for betaine and N-acetylornithine (shown in Figure 6). By discarding each instrument variable in turn and performing MR analysis again, we found that the causal effects of betaine and N-acetylornithine on renal function were not driven by a single SNP. Generally, the causal effects of betaine and N-acetylornithine on renal function or CKD were robust because statistical significances (*p* < 0.05) were still observed in two additional MR tests, the Weighted Median (WM) test and the MR-PRESSO test. The association of betaine on CKD showed non-significant in the Weighted Median test (*p* = 0.15), but it still could be considered as a causal association as the significance observed in IVW method (*p* = 0.0031) and MR-PRESSO test (*p* = 0.0073).

### Reverse MR analysis

We performed reverse MR analysis to rule out the possibility of causal effects of renal function on betaine and N-acetylornithine. The results were shown in Table 2. Consistently, all the exposures showed no causal association with betaine and N-acetylornithin in the IVW method, MR-PRESSO test and WM test (*p* > 0.05), except the association of CKD on betaine in the weighted median test (*p* = 0.038).

**Table 2:** Reverse MR analysis of renal function phenotype on the identified metabolites.

Metabolite	Exposure	SNP number	Method	*p* value	OR (95% CI)	
Betaine	Creatinine-based log-eGFR values (CKDGen + UKB)	161	IVW(fixed)	0.27	0.891 (0.726–1.095)	P_Q_IVW_ = 0.22
		161	WM1	0.33	0.864 (0.644–1.160)	
		161	WM2	0.57	0.811 (0.396–1.659)	
		161	MR Egger	0.45	0.748 (0.354–1.576)	P_intercept_Egger_ = 0.63
		161	MR Presso	0.28	0.891 (0.725–1.096)	P_global test_ = 0.22
	Creatinine-based log-eGFR values (CKDGen)	97	IVW(fixed)	0.99	1.001 (0.809–1.237)	P_Q_IVW_ = 0.61
		97	WM1	0.49	1.115 (0.817–1.523)	
		97	WM2	0.64	1.176 (0.592–2.335)	
		97	MR Egger	0.46	0.699 (0.270–1.814)	P_intercept_Egger_ = 0.45
		97	MR Presso	0.99	1.001 (0.813–1.232)	P_global test_ = 0.65
	CKD (CKDGen)	13	IVW(fixed)	0.077	1.018 (0.998–1.039)	P_Q_IVW_ = 0.68
		13	WM1	0.038	1.030 (1.002–1.059)	
		13	WM2	0.12	1.032(0.994–1.071)	
		13	MR Egger	0.58	1.038 (0.914–1.177)	P_intercept_Egger_ = 0.77
		13	MR Presso	0.067	1.018 (0.999–1.038)	P_global test_ = 0.70
N-acetylornithine	Creatinine-based log-eGFR values (CKDGen + UKB)	161	IVW(random)	0.15	0.735 (0.483–1.118)	P_Q_IVW_ = 0.0077
		161	WM1	0.030	0.533 (0.301–0.941)	
		161	WM2	0.058	0.317 (0.097–1.032)	
		161	MR Egger	0.032	0.183 (0.039–0.849)	P_intercept_Egger_ = 0.067
		161	MR Presso	0.15	0.735 (0.482–1.120)	P_global test_ = 0.052
	Creatinine-based log-eGFR values (CKDGen)	97	IVW(fixed)	0.37	0.827 (0.545–1.256)	P_Q_IVW_ = 0.21
		97	WM1	0.93	0.974 (0.537–1.766)	
		97	WM2	0.77	1.226 (0.311–4.834)	
		97	MR Egger	0.018	0.102 (0.016–0.664)	P_intercept_Egger_ = 0.057
		97	MR Presso	0.38	0.827 (0.544–1.258)	P_global test_ = 0.22
	CKD (CKDGen)	13	IVW(fixed)	0.27	1.304 (0.814–2.088)	P_Q_IVW_ = 0.80
		13	WM1	0.30	1.031 (0.973–1.091)	
		13	WM2	0.63	1.017 (0.951–1.088)	
		13	MR Egger	0.50	0.333 (0.015–7.285)	P_intercept_Egger_ = 0.40
		13	MR Presso	0.29	1.304 (0.797–2.134)	P_global test_ = 0.84

WM1, Weighted Median; WM2, Weighted Mode.

### Genetic correlation analysis

The LDSC analysis found that N-acetylornithine showed a genetic correlation with creatinine-based log-eGFR values provided by CKDGen and UKB(r_g_ = −0.49, *p* = 0.019) and creatinine-based log-eGFR values provided by CKDGen(r_g_ = −0.47, *p* = 0.033) but no statistically significance with CKD outcome provided by CKDGen(r_g_ = 0.67, *p* = 0.072). No genetic correlations was found between betaine and renal function due to negative heritability (as shown in Table 3).

**Table 3:** The genetic correlations between the identified metabolites and renal function phenotype.

Outcome	Metabolite	r_g_	se	z	p
Creatinine-based log-eGFR values (CKDGen + UKB)	Betaine	Negative heritability	/	/	/
	N-acetylornithine	−0.49	0.21	−2.35	0.019
Creatinine-based log-eGFR values (CKDGen)	Betaine	Negative heritability	/	/	/
	N-acetylornithine	−0.47	0.22	−2.13	0.033
CKD (CKDGen)	Betaine	Negative heritability	/	/	/
	N-acetylornithine	0.67	0.37	1.79	0.072

r_g_, genetic correlation; se, standard error; *z*, Z-score; *p*, *p* value for r_g_.

### Multivariable MR analysis

We performed MVMR analysis to adjust for interactions of genetic variant between betaine and N-acetylornithine. Details of the results were shown in Table 4. Both betaine and N-acetylornithine showed causal associations with CKD outcome provided by CKDGen (IVW method: OR (95% CI) = 0.533(0.388–0.733), *p* = 0.00011 for betaine and OR (95% CI) = 1.227(1.096–1.375), *p* = 0.00039 for N-acetylornithine; MR-Lasso feature: OR (95% CI) = 0.579(0.419–0.799), *p* = 0.00089 for betaine and OR (95% CI) = 1.222(1.091–1.369), *p* = 0.00052 for N-acetylornithine; MR-PRESSO: OR (95% CI) = 0.533(0.368–0.772), *p* = 0.00086 for betaine and OR (95% CI) = 1.227(1.078–1.398), *p* = 0.0020 for N-acetylornithine).

**Table 4:** Multivariable MR analysis of the identified metabolites on CKD.

Outcome	Method	Metabolite	*p* value	OR (95% CI)	
CKD (CKDGen)	IVW(fixed)	Betaine	0.00011	0.533 (0.388–0.733)	SNP number = 35
		N-acetylornithine	0.00039	1.227 (1.096–1.375)	P_Heterogeneity test_ = 0.29
	MR-Lasso	Betaine	0.00089	0.579 (0.419–0.799)	SNP number = 34
		N-acetylornithine	0.00052	1.222 (1.091–1.369)	
	MR-Presso	Betaine	0.00086	0.533 (0.368–0.772)	P_Global Test_ = 0.41
		N-acetylornithine	0.0020	1.227 (1.078–1.398)	

### Validation of the causal effects of the identified metabolites on renal function phenotype using cystatin-C-based eGFR

**Figure 7:**
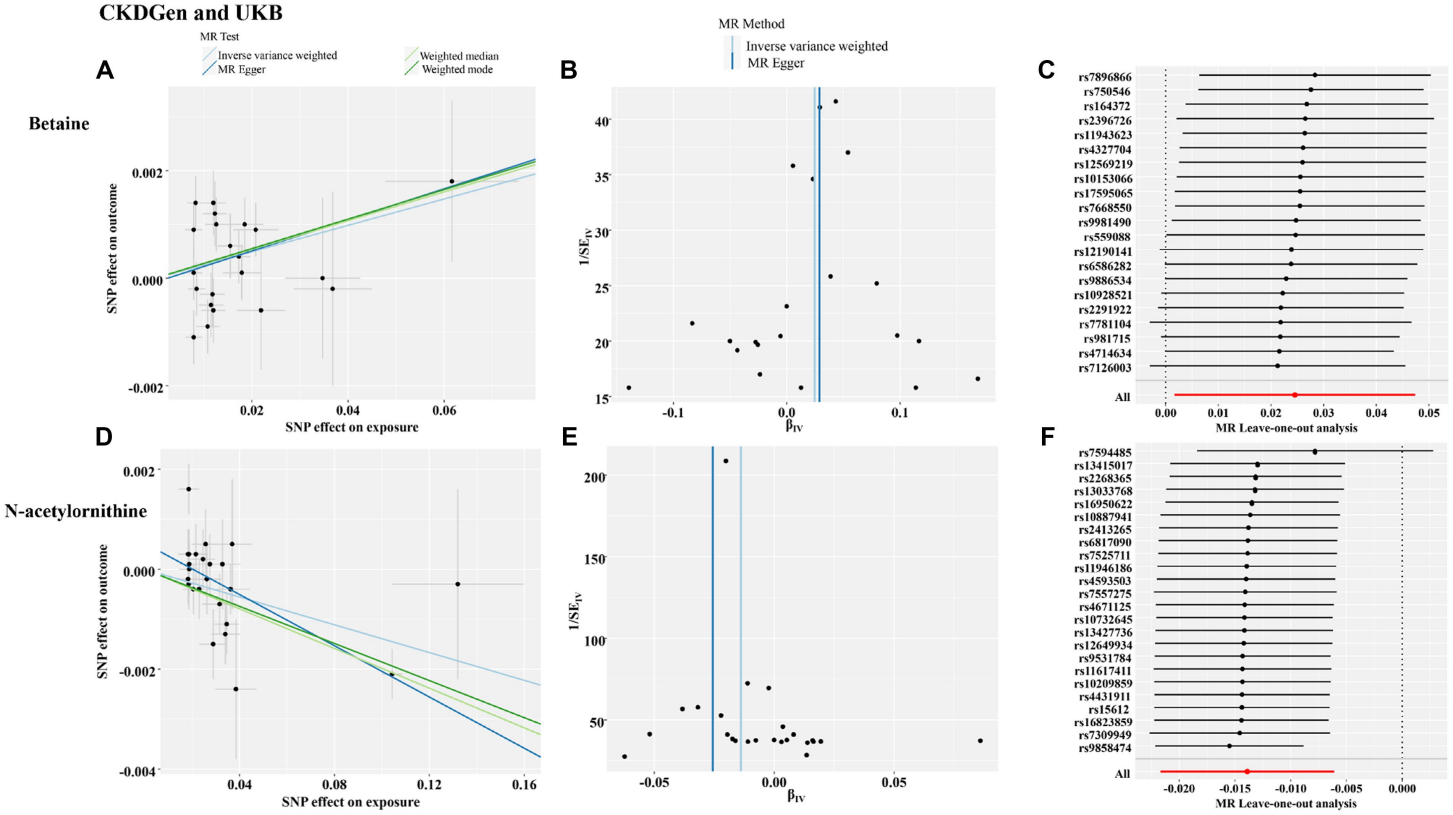
The funnel plot, scatter plot and leave-one-out analysis of the causal interactions of the identified metabolites with cystatin-C-based eGFR from CKDGen and UKB. **(A)** The funnel plot of betaine; **(B)** the scatter plot of betaine; **(C)** leave-one-out analysis of betaine; **(D)** the funnel plot of N-acetylornithine; **(E)** the scatter plot of N-acetylornithine; **(F)** leave-one-out analysis of N-acetylornithine.

To further validate the associations between identified metabolites and renal function, we further assessed causal effects of betaine and N-acetylornithine on cystatin-C-based log-eGFR values. As shown in Table 5, betaine and N-acetylornithine were also found to have suggestive associations with cystatin-C-based eGFR (IVW: OR (95% CI) =1.025 (1.002–1.049), *p* = 0.035 for betaine and OR (95% CI) =0.986 (0.978–0.994), *p* = 0.00046 for N-acetylornithine). Sensitivity analysis showed the robustness of the causal effects using MR-Egger’s intercept term (*p* = 0.86 for betaine; *p* = 0.066 for N-acetylornithine), MR-PRESSO’s global test (*p* = 0.56 for betaine; *p* = 0.12 for N-acetylornithine). Scatter and funnel plots as well as leave-one-out analysis were displayed in Figure 7. Two additional MR tests including the Weighted Median (WM) test and the MR-PRESSO test also showed statistically significant (WM: OR (95% CI) =1.027(1.002–1.053), *p* = 0.037 for betaine and OR (95% CI) =0.980 (0.972–0.989), *p* = 0.000018 for N-acetylornithine; MR-PRESSO: OR (95% CI) =1.025(1.002–1.049), *p* = 0.048 for betaine and OR (95% CI) =0.986(0.979–0.994), *p* = 0.0019 for N-acetylornithine).

**Table 5:** The causal effects of the identified metabolites on cystatin-C-based eGFR from CKDGen and UKB for validation analysis.

Outcome	Metabolite	SNP number	Method	*p* value	OR(95% CI)	
Cystatin-C-based log-eGFR values (CKDGen + UKB)	Betaine	21	IVW(random)	0.035	1.025 (1.002–1.049)	P_Q_IVW_ = 0.011
		21	WM1	0.037	1.027 (1.002–1.053)	
		21	WM2	0.073	1.028 (0.999–1.057)	
		21	MR Egger	0.31	1.029 (0.975–1.087)	P_intercept_Egger_ = 0.86
		21	MR Presso	0.048	1.025 (1.002–1.049)	P_global test_ = 0.056
Cystatin-C-based log-eGFR values (CKDGen + UKB)	N-acetylornithine	24	IVW(fixed)	0.00046	0.986 (0.978–0.994)	P_Q_IVW_ = 0.10
		24	WM1	0.000018	0.980 (0.972–0.989)	
		24	WM2	0.00030	0.982 (0.973–0.990)	
		24	MR Egger	0.00049	0.975 (0.963–0.987)	P_intercept_Egger_ = 0.066
		24	MR Presso	0.0019	0.986 (0.979–0.994)	P_global test_ = 0.12

WM1, Weighted Median; WM2, Weighted Mode.

## Discussion

In this study, we assessed the causal associations between metabolites and renal function using GWAS statistics. We found significant causal effects of betaine and N-acetylornithine on renal function. Higher genetically predicted levels of betaine were significantly associated with higher eGFR and lower incidence of CKD in the three population-scale genetic datasets for main analysis and one additional dataset for validation, consistent with the results of the different MR methods and various MR sensitivity analyses. On the contrary, higher genetically predicted levels of N-acetylornithine were significantly associated with lower eGFR and higher incidence of CKD. MVMR analysis further confirmed the causal effects of betaine and N-acetylornithine on CKD development. N-acetylornithine showed a possible genetic correlation with eGFR using LDSC analysis. Our study may shed new light on the role of gene–environment interactions in the pathogenesis of renal function impairment and provide potential inspiration for further precision treatments.

Blood and urine are typical sample sources for metabolomic identification. Blood is recognized as a good source because it contains numerous detectable metabolites and can easily be obtained. Thus, metabolites have the potential to be biomarkers of treatment and disease progression. In the recent decade, metabolomics has been applied to explore the relationship between blood metabolomic profiling and kidney disease (17). Several metabolic studies indicated that some metabolites such as tiglylcarnitine, kynurenine, arginine metabolites and tryptophan-related metabolism identified from serum or urine samples may be related with progression of CKD (4, 5, 18). But it is not easy to determine the causal association using cress-sectional or longitudinal observational studies due to high costs for measurements of all blood metabolites and the difficulty in explaining the intrinsic relationships. In this regards, Mendelian randomization may be a good tool to interpret the causal effects of metabolites on renal function. The present study was the first of its kind to investigate the causal effects of metabolites on renal function based on metabolomics. Moreover, it provided the first evidence for the causal associations between renal function and two identified metabolites using several population-scale genetic datasets.

Betaine is a methyl group donor involved in the remethylation of homocysteine to produce methionine. It acts as a cell-compatible osmolyte which plays an essential role in regulation of osmotic pressure and is important for maintenance of enzymatic functions, cell metabolism stability, and electrical signaling (19). In addition, betaine was reported to inhibit NF-κB, an essential activator of the inflammatory response, possibly through the suppression of mitogen-activated protein kinases and the IκB kinase (IKK) complex (20, 21). It could also suppress the NLR family pyrin domain containing 3 (NLRP3) inflammasome by inhibiting mature caspase-1 and IL-1β formation (22, 23). What’s more, betaine could increase the production of antioxidants such as SOD and CAT and subsequently ameliorated the oxidative stress (24). Thus, betaine may help to protect against consistent inflammation and excessive oxidative stress, which play an important role in renal fibrosis and renal function impairment. Previous reports suggested that plasma levels of betaine in patients with CKD decreased with progression of renal failure, whereas patients with end stage renal disease (ESRD) had lower plasma betaine levels compared with early-stage patients and healthy volunteers (25, 26). This was consistent with our findings. The association between betaine intake and renal function has not been deeply studied. A cross-over controlled trial with small sample size indicated that supplementation of betaine for 3 months decreased plasma homocysteine concentrations after methionine loading in CKD patients (27). Recently a randomized controlled trial found that a mediterranean dietary pattern with high intake of betaine from fish, eggs, vegetables, nuts, and whole-grain cereals may improve some renal parameters such as urine albumin creatinine ratio (UACR) (28). In this study, we also found causal effects of betaine on renal function estimated by both creatinine-based and cystatin-C-based eGFR through application of genetic variants, which further supported the idea that betaine may protect against impairment of renal function with the use of population-scale datasets for the first time. Notably, in addition to the protective roles of betaine, recent studies also pointed out that betaine was one of the precursors of trimethylamine N-oxide (TMAO), a pro-atherogenic metabolite (29). For CKD patients, gut dysbiosis was reported to result in an abnormal metabolism of betaine and TMAO and lead to excessive production of TMAO, which possibly increased the mortality of CKD patients from cardiovascular causes (30). It remains unclear whether supplementation of betaine would affect plasma TMAO concentrations. Considering the findings that increased betaine intake due to increased consumption of fruits and decreased consumption of refined-grain cereals and red meat gave rise to improvement of cardiometabolic parameters (31), the food source of betaine should be valued and supplementation of betaine may be potential treatment for CKD patients to ameliorate renal function worsening regardless of concerns about TMAO.

N-acetylornithine is the production of arginine metabolism. A previous study found that plasma levels of N-acetylornithine were significantly higher in type 2 diabetes patients with diabetic nephropathy compared with type 2 diabetes patients without diabetic nephropathy (32). Consistently, another investigation showed negative correlation between circulating levels of N-acetylornithine and eGFR based on several African American cohorts with relatively small sample size (33). But the causal effect of N-acetylornithine on renal function remained unclear. In this study, through Mendelian randomization, we found that increased plasma levels of N-acetylornithine may contribute to the impairment of renal function based on four population-scale datasets. It deserves further investigations whether N-acetylornithine could be employed as a causal determinant or treatment target of CKD progression. Function research and follow-up cohort studies with large sample were likely to be required to bridge the gap between this statistical causation and clinical translation.

The present study had several strengths. First, the most significant strength of this study was the large scale of genetic variables we covered to analyze the relationship between blood metabolites and renal function. A total of 309 metabolites were covered after exclusion of unidentified metabolites. Meanwhile, four GWAS datasets for the genetic variables of renal function were applied including up to 1,004,040 individuals classified by creatinine-based eGFR or cystatin-C-based eGFR. Benefiting from these GWAS datasets, we made our study a relatively comprehensive and systematic analysis of the metabolic profile related to renal function impairment. Second, using the MR design including reverse MR analysis, our study avoided reverse causality and residual confounding factors. Additional sensitivity analysis and multivariable MR analysis were undertaken to confirm the robustness of our study. Third, previous MR studies on renal function mainly assessed the causal effect of gut microbiota and its associated metabolites on renal function. Our study was the first MR study to systematically explore the causal effects of blood metabolites on renal function based on metabolomics.

There were still some limitations in our study. First, we only estimated the linear effect of metabolites on renal function but we could not exclude the possibility of a nonlinear effect for some metabolites. Second, the analysis were mainly based on European ancestry samples, which limited the cross-ethnic extrapolation of our findings. Third, since the overall effect sizes in the results were small especially when the outcome was log-eGFR values, a rapid and dramatic change of metabolites may have different effects on renal function. Fourth, the functions and mechanisms of the identified metabolites in the disease were not fully elucidated, which restricted our interpretation of the findings from this MR analysis.

In conclusion, this was the first systematic metabolome-wide MR analysis to estimate the causal relationship between blood metabolites and renal function using GWAS datasets. We found out some candidate metabolites with potential effects on renal function in various datasets. Betaine and N-acetylornithine were identified as the causal metabolites which passed main MR analysis and sensitivity analysis in all the datasets. This provided preliminary evidence on the impact of such metabolites levels on the risk of renal function impairment. Further studies may be warranted for biomarker validation, mechanism exploration and drug target selection of these identified metabolites.

## Data availability statement

Publicly available datasets were analyzed in this study. This data can be found here: all data used in this study could be obtained from the metabolomics GWAS server (https://gwas.mrcieu.ac.uk/) and the CKDGen consortium (http://ckdgen.imbi.uni-freiburg.de/datasets).

## Ethics statement

Ethical approval was not required for the study involving humans in accordance with the local legislation and institutional requirements. Written informed consent to participate in this study was not required from the participants or the participants’ legal guardians/next of kin in accordance with the national legislation and the institutional requirements.

## Author contributions

YL: Data curation, Formal analysis, Writing – original draft. LL: Formal analysis, Validation, Writing – original draft. YS: Funding acquisition, Visualization, Writing – original draft. XB: Conceptualization, Funding acquisition, Validation, Visualization, Writing – review & editing.

## Footnotes

^1^ https://gwas.mrcieu.ac.uk/

^2^ https://ldlink.nih.gov/?tab=home

^3^ http://ckdgen.imbi.uni-freiburg.de/datasets

## References

[1] Kalantar-Zadeh K Jafar TH Nitsch D Neuen BL Perkovic V. Chronic kidney disease. Lancet. (2021) 398:786–802. doi: 10.1016/S0140-6736(21)00519-5 34175022

[2] Cho JM Koh JH Kim SG Lee S Kim Y Cho S. Mendelian randomization uncovers a protective effect of interleukin-1 receptor antagonist on kidney function. Commun Biol. (2023) 6:722. doi: 10.1038/s42003-023-05091-8, PMID: 37452175 PMC10349143

[3] Coresh J Inker LA Sang Y Chen J Shafi T Post WS. Metabolomic profiling to improve glomerular filtration rate estimation: a proof-of-concept study. Nephrol Dial Transplant. (2019) 34:825–33. doi: 10.1093/ndt/gfy094, PMID: 29718360 PMC6503300

[4] Yang Y Ma C Li S Cai W Dai W Zhang X. Urinary microbiota and serum metabolite analysis in patients with diabetic kidney disease. Heliyon. (2023) 9:e17040. doi: 10.1016/j.heliyon.2023.e17040, PMID: 37521000 PMC10382294

[5] Balint L Socaciu C Socaciu AI Vlad A Gadalean F Bob F. Metabolites potentially derived from gut microbiota associated with podocyte, proximal tubule, and renal and cerebrovascular endothelial damage in early diabetic kidney disease in T2DM patients. Meta. (2023) 13:893. doi: 10.3390/metabo13080893, PMID: 37623837 PMC10456401

[6] Hui Y Zhao J Yu Z Wang Y Qin Y Zhang Y. The role of tryptophan metabolism in the occurrence and progression of acute and chronic kidney diseases. Mol Nutr Food Res. (2023) 67:e2300218. doi: 10.1002/mnfr.202300218 37691068

[7] Smith GD Ebrahim S. 'Mendelian randomization': can genetic epidemiology contribute to understanding environmental determinants of disease? Int J Epidemiol. (2003) 32:1–22. doi: 10.1093/ije/dyg070, PMID: 12689998

[8] Kettunen J Demirkan A Wurtz P Draisma HH Haller T Rawal R. Genome-wide study for circulating metabolites identifies 62 loci and reveals novel systemic effects of LPA. Nat Commun. (2016) 7:11122. doi: 10.1038/ncomms11122, PMID: 27005778 PMC4814583

[9] Davies NM Holmes MV Davey SG. Reading Mendelian randomisation studies: a guide, glossary, and checklist for clinicians. BMJ. (2018) 362:k601. doi: 10.1136/bmj.k601 30002074 PMC6041728

[10] Shin SY Fauman EB Petersen AK Krumsiek J Santos R Huang J. An atlas of genetic influences on human blood metabolites. Nat Genet. (2014) 46:543–50. doi: 10.1038/ng.2982, PMID: 24816252 PMC4064254

[11] Pierce BL Ahsan H Vanderweele TJ. Power and instrument strength requirements for Mendelian randomization studies using multiple genetic variants. Int J Epidemiol. (2011) 40:740–52. doi: 10.1093/ije/dyq151, PMID: 20813862 PMC3147064

[12] Hemani G Zheng J Elsworth B Wade KH Haberland V Baird D. The MR-base platform supports systematic causal inference across the human phenome. eLife. (2018) 7:7. doi: 10.7554/eLife.34408 PMC5976434 29846171

[13] Wuttke M Li Y Li M Sieber KB Feitosa MF Gorski M. A catalog of genetic loci associated with kidney function from analyses of a million individuals. Nat Genet. (2019) 51:957–72. doi: 10.1038/s41588-019-0407-x 31152163 PMC6698888

[14] Stanzick KJ Li Y Schlosser P Gorski M Wuttke M Thomas LF. Discovery and prioritization of variants and genes for kidney function in >1.2 million individuals. Nat Commun. (2021) 12:4350. doi: 10.1038/s41467-021-24491-0, PMID: 34272381 PMC8285412

[15] Bulik-Sullivan B Finucane HK Anttila V Gusev A Day FR Loh PR. An atlas of genetic correlations across human diseases and traits. Nat Genet. (2015) 47:1236–41. doi: 10.1038/ng.3406, PMID: 26414676 PMC4797329

[16] Sanderson E. Multivariable Mendelian randomization and mediation. Cold Spring Harb Perspect Med. (2021) 11:11. doi: 10.1101/cshperspect.a038984 PMC7849347 32341063

[17] Rhee EP. How omics data can be used in nephrology. Am J Kidney Dis. (2018) 72:129–35. doi: 10.1053/j.ajkd.2017.12.008, PMID: 29478865 PMC6019111

[18] Trischitta V Mastroianno M Scarale MG Prehn C Salvemini L Fontana A. Circulating metabolites improve the prediction of renal impairment in patients with type 2 diabetes. BMJ Open Diab Res Care. (2023) 11:11. doi: 10.1136/bmjdrc-2023-003422 PMC10514631 37734903

[19] Ratriyanto A Mosenthin R. Osmoregulatory function of betaine in alleviating heat stress in poultry. J Anim Physiol Anim Nutr. (2018) 102:1634–50. doi: 10.1111/jpn.12990, PMID: 30238641

[20] Go EK Jung KJ Kim JY Yu BP Chung HY. Betaine suppresses proinflammatory signaling during aging: the involvement of nuclear factor-kappaB via nuclear factor-inducing kinase/IkappaB kinase and mitogen-activated protein kinases. J Gerontol Ser A-Biol Sci Med Sci. (2005) 60:1252–64. doi: 10.1093/gerona/60.10.1252 16282556

[21] Lee EK Jang EJ Jung KJ Kim DH Yu BP Chung HY. Betaine attenuates lysophosphatidylcholine-mediated adhesion molecules in aged rat aorta: modulation of the nuclear factor-kappaB pathway. Exp Gerontol. (2013) 48:517–24. doi: 10.1016/j.exger.2013.02.024, PMID: 23466300

[22] Li Y Pan L He YC. Co-production of 2,5-dihydroxymethylfuran and furfuralcohol from sugarcane bagasse via chemobiocatalytic approach in a sustainable system. Bioresour Technol. (2023) 389:129819. doi: 10.1016/j.biortech.2023.129819 37797802

[23] Xia Y Chen S Zhu G Huang R Yin Y Ren W. Betaine inhibits interleukin-1beta production and release: potential mechanisms. Front Immunol. (2018) 9:2670. doi: 10.3389/fimmu.2018.02670, PMID: 30515160 PMC6255979

[24] Li C Wang Y Li L Han Z Mao S Wang G. Betaine protects against heat exposure-induced oxidative stress and apoptosis in bovine mammary epithelial cells via regulation of ROS production. Cell Stress Chaperones. (2019) 24:453–60. doi: 10.1007/s12192-019-00982-4, PMID: 30805833 PMC6439124

[25] Guo F Dai Q Zeng X Liu Y Tan Z Zhang H. Renal function is associated with plasma trimethylamine-N-oxide, choline, L-carnitine and betaine: a pilot study. Int Urol Nephrol. (2021) 53:539–51. doi: 10.1007/s11255-020-02632-6, PMID: 32945995

[26] Missailidis C Hallqvist J Qureshi AR Barany P Heimburger O Lindholm B. Serum trimethylamine-N-oxide is strongly related to renal function and predicts outcome in chronic kidney disease. PLoS One. (2016) 11:e141738. doi: 10.1371/journal.pone.0141738 PMC4709190 26751065

[27] McGregor DO Dellow WJ Robson RA Lever M George PM Chambers ST. Betaine supplementation decreases post-methionine hyperhomocysteinemia in chronic renal failure. Kidney Int. (2002) 61:1040–6. doi: 10.1046/j.1523-1755.2002.00199.x, PMID: 11849459

[28] Diaz-Lopez A Becerra-Tomas N Ruiz V Toledo E Babio N Corella D. Effect of an intensive weight-loss lifestyle intervention on kidney function: a randomized controlled trial. Am J Nephrol. (2021) 52:45–58. doi: 10.1159/000513664, PMID: 33556935

[29] Yang S Li X Yang F Zhao R Pan X Liang J. Gut microbiota-dependent marker TMAO in promoting cardiovascular disease: inflammation mechanism, clinical prognostic, and potential as a therapeutic target. Front Pharmacol. (2019) 10:1360. doi: 10.3389/fphar.2019.01360, PMID: 31803054 PMC6877687

[30] Xu KY Xia GH Lu JQ Chen MX Zhen X Wang S. Impaired renal function and dysbiosis of gut microbiota contribute to increased trimethylamine-N-oxide in chronic kidney disease patients. Sci Rep. (2017) 7:1445. doi: 10.1038/s41598-017-01387-y, PMID: 28469156 PMC5431124

[31] Diez-Ricote L San-Cristobal R Concejo MJ Martinez-Gonzalez MA Corella D Salas-Salvado J. One-year longitudinal association between changes in dietary choline or betaine intake and cardiometabolic variables in the PREvencion con DIeta MEDiterranea-plus (PREDIMED-plus) trial. Am J Clin Nutr. (2022) 116:1565–79. doi: 10.1093/ajcn/nqac255, PMID: 36124652 PMC9761742

[32] Devi S Nongkhlaw B Limesh M Pasanna RM Thomas T Kuriyan R. Acyl ethanolamides in diabetes and diabetic nephropathy: novel targets from untargeted plasma metabolomic profiles of south Asian Indian men. Sci Rep. (2019) 9:18117. doi: 10.1038/s41598-019-54584-2, PMID: 31792390 PMC6889195

[33] Luo S Surapaneni A Zheng Z Rhee EP Coresh J Hung AM. NAT8 variants, N-acetylated amino acids, and progression of CKD. Clin J Am Soc Nephrol. (2020) 16:37–47. doi: 10.2215/CJN.08600520 33380473 PMC7792648

